# The genetic characterization of grapevines prospected in old Serbian vineyards reveals multiple relationships between traditional varieties of the Balkans

**DOI:** 10.3389/fpls.2024.1391679

**Published:** 2024-07-11

**Authors:** Javier Tello, Slavica Todić, Yolanda Ferradás, Miroslav Nikolic, Aneta Sabovljević, Dragoslav Ivanišević, Željko Tomanović, Miodrag Grbić, José Miguel Martínez-Zapater, Javier Ibáñez

**Affiliations:** ^1^ Departamento de Viticultura, Instituto de Ciencias de la Vid y del Vino [ICVV, CSIC-Gobierno de La Rioja-Universidad de La Rioja (CSIC-CAR-UR)], Logroño, Spain; ^2^ Faculty of Agriculture, Department of Viticulture, University of Belgrade, Belgrade, Serbia; ^3^ Institute for Multidisciplinary Research, University of Belgrade, Belgrade, Serbia; ^4^ Faculty of Biology, University of Belgrade, Belgrade, Serbia; ^5^ Faculty of Agriculture, University of Novi Sad, Novi Sad, Serbia; ^6^ Serbian Academy of Sciences and Arts, Belgrade, Serbia; ^7^ Department of Biology, University of Western Ontario, London, ON, Canada; ^8^ Center for Interdisciplinary and Multidisciplinary Studies, University of Montenegro, Podgorica, Montenegro

**Keywords:** grape cultivar, pedigree, SNP, SSR, *Vitis vinifera* L., Western Balkans

## Abstract

Serbia preserves a high number of local grape varieties, which have been cultivated across the country for centuries. Now, these ancient varieties are in the spotlight, and there is a global trend towards their recovery and characterization because they can revitalize regional, national and international grape and wine sectors. In addition, their genetic study can be useful to find new pedigree relationships to reveal how local varietal assortment evolved over time. Here, the genetic characterization of 138 grapevines from old Serbian vineyards revealed 59 different genetic profiles, 49 of which were identified as grapevine varieties whose origin in the country could be linked to some major Serbian historical periods. Most of the genetic profiles found in this work arranged in a complex pedigree network that integrates numerous grapevine varieties from diverse Balkan countries, agreeing with an intense exchange of plant material among Balkan regions for centuries. This analysis identified some varieties as important founders of Balkan genetic resources, like ‘Alba Imputotato’, ‘Braghina Rosie’, ‘Coarna Alba’, and ‘Vulpea’. After deepening into their genealogy, these major direct founders might have ultimately derived from ‘Visparola’, an ancient variety of likely Balkan origin with a major founding role in some European regions. Our results also indicated the genetic singularity of the grapevine resources from the Balkans when compared to those from other relevant winemaking regions, supporting the interest of their detailed study to evaluate their oenological potential and for the eventual identification of useful traits to counteract current viticulture challenges.

## 1 Introduction

The favourable climate and geological characteristics of the Southern Balkans prompted grapevine (*Vitis vinifera* L.) cultivation since ancient times, with some of the earliest evidences of winemaking activities traced back to the Bronze and Iron Ages (Ladoukakis et al., 2005; Bešlić et al., 2012; Žulj Mihaljević et al., 2015; Maraš et al., 2020). Recent genomic studies suggest that Balkan grapevine varieties diversified from some early domesticated forms originated in Western Asia, which were brought and dispersed in the region about 8,000 years ago (Dong et al., 2023). These firstly introduced domesticates likely introgressed with original local wild grapevines in a progressive process giving rise to a distinctive pool of local grapevines (Magris et al., 2021; Dong et al., 2023). Then, some of these ancient local varieties could disperse to Western regions, in a stepwise diversification process that contributed to Central, Western European, and Iberian wine grapes (Dong et al., 2023). So, considering the natural and human-driven terrestrial dispersion of vines, and its geographical situation, the Balkan Peninsula can be considered as a “bridge region” between the original domestication sites and the regions of origin of some of today’s most important wine grape varieties. According to their geographical origin and morphological features, *V. vinifera* L. varieties are traditionally classified into three *proles*: *occidentalis*, *orientalis*, and *pontica* (Negrul, 1946), a major division supported by molecular data (Bacilieri et al., 2013; Emanuelli et al., 2013; Laucou et al., 2018). Whilst extensive works analyse the genetic diversity of varieties from the *proles occidentalis* (wine varieties from Western Europe) and *orientalis* (Eastern table and raisin grape varieties, and Muscats), little is known on those from the *proles pontica*. The uniqueness of the Balkan grapevine varieties was already pointed out by the botanist Negrul, who also suggested a subdivision of the proles *pontica* into two *subproles*: *georgica* and *balcanica* (Negrul, 1946). As its name indicates, the *subproles balcanica* distinctively groups numerous varieties from the Balkans, including varieties growing in Serbia like ‘Prokupac’, ‘Smederevka’ (syn. ‘Dimyat’), and ‘Začinak’ (Bešlić et al., 2012).

Situated in the heart of the Balkan Peninsula, Serbia’s terrain ranges from the flat plains and hilly slopes of the Fruška Gora Mountains in the northern part of the country (Vojvodina), to the undulating terrains in the east (Negotinska Krajina), south (Župa and Toplica), and central (Šumadija) regions, providing a spectrum of terroirs and climates conducive to successful grape cultivation. Evidence of wine production and consumption in Serbia dates back to the Bronze and Iron Ages, with a notable influence from the Ancient Greeks, Romans, and Medieval times (Bešlić et al., 2012). Like other Balkans countries, Serbia holds a high number of local grapevine varieties, which have been cultivated across the country for a long time (Bešlić et al., 2012; Dabić Zagorac et al., 2019). However, diverse reports indicate that current varietal assortment was shortened after some historic events, which shaped the extent of genetic diversity that exists today. For example, the cultivation of table grapes for fresh consumption and raisins was favoured under the Ottoman influence (14^th^ to 19^th^ centuries), which in turn reduced the cultivation of grapes for winemaking (Bešlić et al., 2012). This trend caused the introduction of table grape varieties from Eastern regions, as observed in neighbouring regions (Carka et al., 2015; Maraš et al., 2020). Likewise, and as in other European winemaking regions, the expansion of different disease-causing agents at the end of the 19^th^ century caused the irreversible disappearance of some local grapevines (Bešlić et al., 2012). Genetic diversity was specially affected by the effect of grape phylloxera, which was already observed in vineyards of the Serbian region of Smederevo in 1882 (Garić Petrović, 2020), soon after a major outbreak happened in the city of Klosterneuburg (Austria) in 1868 (Tello et al., 2019). Many local vineyards fell upon the action of this pest, which led to the loss of some local varieties. After this crisis, devastated vineyards were replanted with grafted vines (Garić Petrović, 2020), commonly replacing local varieties by foreign varieties to accommodate to international market trends. Today, the Serbian wine industry still relies on the cultivation of a reduced number of international varieties, like ‘Cabernet Sauvignon’, ‘Merlot’, ‘Chardonnay’, and ‘Sauvignon Blanc’, and autochthonous varieties like ‘Prokupac’ represent a minor fraction of the Serbian vineyard (Milišić et al., 2021). Nevertheless, there is a raising interest in the identification and promotion of the cultivation of indigenous and regional varieties like ‘Smederevka’ (syn. ‘Dimyat’), ‘Začinak’ and ‘Bagrina’ (syn. ‘Braghina Rosie’) as an opportunity of diversification and distinction in an increasingly demanding and crowded global wine sector.

Grapevine is one of the most important fruit crops in the world, with an annual production of *ca*. 78 mt grapes that sustains worldwide wine elaboration and fresh grape, unfermented juice, and dried grape markets (www.oiv.net). Although overall grape production relies on the cultivation of a reduced number of varieties (like ‘Cabernet Sauvignon’, ‘Merlot Noir’, or ‘Tempranillo Tinto’), there are between 6,000 and 10,000 different genotypes worldwide, each one with unique features (Wolkovich et al., 2018). Nowadays, traditional viticulture systems’ sustainability is compromised by different threats, like those derived from climate change (Delrot et al., 2020). In this regard, *V. vinifera* L. natural diversity can be used as a tool to face most of these challenges (Morales-Castilla et al., 2020). Multiple reports indicate the presence of a high level of “hidden diversity” in old and/or abandoned vineyards of poorly explored regions, which might be key to solve current and future problems (Žulj Mihaljević et al., 2015; Maraš et al., 2020; Sargolzaei et al., 2021; Zombardo et al., 2021). As demonstrated in different works (Balda et al., 2014; Maraš et al., 2020; Zombardo et al., 2021; Barrias et al., 2023), these sites are considered as reservoirs of genetic diversity, where many factors (climate, soil conditions, human dedication) led to the preservation of different varieties. Thus, the prospection and characterization of genetic resources from these vineyards might result in the recovery of old varieties with some putative relevance in the past, which now might be important to face current and future viticulture challenges (Maraš et al., 2020; Zombardo et al., 2021). The study of these genetic resources can also provide useful information to know how traditional viticultural systems evolved (Žulj Mihaljević et al., 2015; Maraš et al., 2020). In addition, it can lead to discover some original genotypes that have not yet been collected or referenced, which can reveal the genetic origin of some relevant varieties (Boursiquot et al., 2009; Ibáñez et al., 2012). To this aim, the combined study of nuclear microsatellites (Simple Sequence Repeats, SSRs), nuclear single nucleotide polymorphisms (SNPs), and chloroplast SNPs have proved to be a powerful strategy for the genetic characterization of grapevine varieties of different regions (Cunha et al., 2020; Maraš et al., 2020; Nebish et al., 2021). These genetic markers provide a high level of information for diversity, phylogenetic, and pedigree studies (Cunha et al., 2020; Maraš et al., 2020; Barrias et al., 2023), as well as to deep into the history of grapevine cultivation (Bacilieri et al., 2013; Emanuelli et al., 2013; Laucou et al., 2018).

In this work, we report the results of the genetic identification of 138 grapevines from the historically most relevant winemaking regions of Serbia, most of them prospected from vineyards over 50 years old. Also, some isolated vines that survived the clearing of old vineyards have been included. These vines were found in meadows and edges of forests that have taken over the areas where old vineyards were once located. Their screening at 7 SSR and 240 SNP *loci* revealed 59 different genetic profiles, some of them not referenced yet. The analysis of these genetic profiles allowed to discover the full pedigree of some relevant Balkan varieties. In addition, this information revealed multiple relationships between grapevines from the Balkans and beyond, somehow reflecting the history of the region, and it pinpointed some varieties as major founders of local genetic resources. To our knowledge, this is the largest study focused on autochthonous grapevine varieties from Serbia performed so far.

## 2 Materials and methods

### 2.1 Plant material

After several inspections of old vineyards (in general, over 50 years old) conducted in 2020 and 2021 from the main winegrowing regions of Serbia, 138 grapevines were selected for genetic analyses (**Supplementary Material 1**). This set of samples included 50 grapevines from Vojvodina (North Serbia), 38 from East Serbia, 13 from Central Serbia, one from West Serbia and 36 from South Serbia. The selected vines were tagged, photodocumented, and GPS referenced. When possible, local names given by grape growers and the estimated age of the vineyards were noted too. Young leaves (about 5 cm in length) were collected *in situ* from each plant, and stored in ice until its final storage at -80°C.

### 2.2 DNA extraction and genotyping

Grapevine young leaf samples (100 mg fresh weight) were grounded into powder in individual mortars using liquid nitrogen. Then, whole genomic DNA (gDNA) was extracted from 100 mg of ground leaf powder using the NZY Plant gDNA isolation kit (NZYTech, Lisbon, Portugal), as recommended by the manufacturer. gDNA quality and quantity was evaluated using a spectrophotometer (Nanodrop, Thermo Scientific, Wilmington, USA). All samples were genotyped at seven highly polymorphic SSR markers (*VVMD5*, *VVMD7*, *VVMD27*, *VVMD32*, *VVS2*, *VrZAG62*, and *VrZAG79*) in a single multiplex polymerase chain reaction (PCR), as indicated in Nebish et al. (2021). This set of markers include the six SSR markers recommended for grapevine identification (This et al., 2004; Maul et al., 2012). Fragments were then subjected to capillary electrophoresis, which was performed in the genotyping platform of the Centro de Investigación Biomédica de La Rioja (CIBIR). To this aim, PCR products were mixed with 20 μl of highly deionized (Hi-Di) formamide and 0.2 μl of GeneScan-500 LIZ size standards (both from Applied Biosystems, Foster City, CA, USA), and denaturalized at 95°C for 5 min. Fragment sizes were then rated by means of GeneMapper v.4.1 (Applied Biosystems, Darmstadt, Germany). Each analysis included one DNA of ‘Tempranillo Tinto’ as positive control, and a non-template tube as a negative control.

Non redundant grapevine samples were also screened at the 240 nuclear SNP *loci* described by Zinelabidine et al. (2012), using Fluidigm technology. This analysis was done in the Sequencing and Genotyping Unit of the Universidad del País Vasco (UPV/EHU). This set of markers includes a core set of 48 SNPs for variety identification (Cabezas et al., 2011), and an extended set of 192 SNPs for parentage and diversity analyses (Zinelabidine et al., 2012). It also includes five chloroplast SNPs that allows the differentiation of the four most common grapevine chloroplast haplotypes (chlorotypes A, B, C and D) described by Arroyo-García et al. (2006).

### 2.3 Varietal identification

Non-redundant genetic profiles were compared to those available at the *Vitis* International Variety Catalogue (*V*IVC, 01/11/2023, 6,354 SSR profiles available) and at the ICVV-DNA database (01/11/2023, 3,574 SNP and/or SSR profiles available) for varietal identification. Genetic profiles with no matching profile in these two databases were considered as unknown.

### 2.4 Genetic and parentage analyses

The non-redundant genetic profiles obtained in this study for the set of 240 SNPs were merged with those available at the ICVV-DNA database for a wide search of possible first-order kinship relationships (trios mother-father-offspring and duos parent-offspring). To this aim, the likelihood-based method implemented in Cervus v.3.0 (Kalinowski et al., 2007) was used essentially as described in Cunha et al. (2020). Parent pair (trio) analysis in Cervus was performed in two steps to avoid common computational constraints occurring when a large number of genotypes is used as candidate parents against all offspring candidates. First, a paternity analysis was carried out to get all possible duos for every genotype with 5 or less mismatching (non-compatible) SNP. This resulted in a reduced number of candidates for each offspring, which was used in a second step to prepare a separate list of candidate parents for each offspring, for the actual parent pair (trio) analysis. The natural logarithm of the overall likelihood ratio (LOD) score was used to rate the robustness of each detected relationship. For trios, the maximum number of mismatching SNPs was set to two. For duos, only one mismatching SNP was allowed, and only those with a LOD > 25 were considered. SSR data was used to confirm the relationships indicated by SNP data. Lastly, chlorotype information, when differed between the two parents, was used to determine the female progenitor in the proposed trios, considering the maternal inheritance of chloroplasts in grapevine (Arroyo-García et al., 2006).

Besides, an Unweighted Neighbour-Joining (UwNJ) distance tree was calculated to analyse the relationship between the genotypes identified in this work. Thus, this test focused on the analysis of the genotypes identified as varieties from different Balkan countries and those unidentified putative Balkan varieties that did not match any known genetic profile. For that, we calculated a dissimilarity matrix based on 10,000 bootstrap steps calculated by the DARwin software package v. 6.0.21 (Perrier and Jacquemond-Collet, 2006). Similarly, we analysed the relationship between all these genotypes and a series of representative varieties from three contrasting winemaking regions (as proposed by *V*IVC database): the Caucasus (including varieties from Armenia, Georgia and Azerbaijan), the Iberian Peninsula (Spain and Portugal), and Central Europe (France and Germany) (**Supplementary Material 2**). To this aim, the 240-SNP genetic profile of 90 genotypes (30 per region) were retrieved from the ICVV-DNA database. The R package *adegenet* (Jombart, 2008) was used to perform a discriminant analysis of principal components (DAPC) based on 240-SNPs data. This analysis was complemented by the construction of a second UwNJ distance tree, as detailed before.

## 3 Results

### 3.1 Genetic identification of old grapevine varieties

After the inspection of numerous vineyards across the main winegrowing regions of Serbia, 138 old grapevines were selected for their genetic characterization. SSR and SNP genetic profiling identified up to 59 different genetic profiles (fully provided in the **Supplementary Material 3**), which were compared with the reference genetic profiles stored at the *V*IVC and ICVV-DNA databases for varietal identification. The comparison of the SSR profiles led to the identification of 127 samples, corresponding to 49 known grapevine varieties (**Table 1**; **Supplementary Material 1**). For the remaining 11 samples, 10 different genetic profiles were found, which had no corresponding genetic SSR profile at the *V*IVC database. However, we found a matching SNP genotype for four of these 10 profiles in the ICVV-DNA database, obtained from samples previously analysed in our laboratory from grapevines of the Western Balkans (**Supplementary Material 1**). This indicates the potential local origin of these four unidentified genotypes. The remaining six unidentified genetic profiles have been found only once until to date, so they could be single plants grown from a seed, or actual varieties at an extremely high risk of disappearance.

**Table 1:** Grapevine varieties identified in old Serbian vineyards after the genetic profiling of 138 grapevines at 7 SSR and 240 SNP *loci*.

Variety Name	N	*V*IVC Variety Number	Use ^a,b^	Origin ^a,c^	Chlorotype
Agadai	1	95	T, W	Daghestan	D
Bakator Belyi	1	904	W	SUN	C
Bela Dinka	1	16848	W	Serbia	D
Berbecel	1	1148	W	Bulgaria	D
Blaufraenkisch	1	1459	T, W	Slovenia	C
Braghina Rosie	15	1644	T, W	Serbia	C
Chaouch Blanc	1	10196	T	Turkey	C
Chasselas	1	2473	T, W	–	D
Coarna Alba	3	2724	T, W	Moldova	C
Coarna Neagra	1	2726	T, W	Moldova	B
Csaba Gyoengye	1	9166	T, W	Hungary	C
Dimyat	5	5716	T, W	Bulgaria	C
Ezerjo	1	4027	W	Hungary	C
Ferdinand de Lesseps	4	4088	T, W	United Kingdom	–
Furmint	1	4292	W	Hungary	C
Grk Crni	1	5067	W	Serbia	C
Harslevelue	1	5314	T, W	Hungary	C
Kadarka Feher	1	5899	W	Hungary	C
Kadarka Kek	1	5898	W	Hungary	D
Kadarka SRB	1	24623	W	Serbia	D
Knipperle	1	6312	W	France	A
Koevidinka	1	13727	W	Hungary	C
Kokur Belyi	1	6375	T, W	Ukraine	A
Kreaca	1	6501	T, W	Serbia	C
Krivaja	1	24929	T	Serbia	C
Krkošija	1	16850	W	Serbia	C
Lisztes Feher	1	17061	W	Hungary	C
Moscato Giallo	3	8056	T, W	Italy	A
Muscat a Petits Grains	6	8193	T, W	Greece	D
Muscat Fleur d’Oranger	1	8221	T, W	France	D
Mustoasa de Maderat	1	8311	W	Balkan	C
Negru Virtos	1	8466	W	Romania	C
Ohridsko Belo	1	21403	W	North Macedonia	D
Pamid	29	8899	W	Bulgaria	C
Parmak Cerven	1	8945	T	Turkey	C
Pinot	1	9279	W	France	A
Prokupac	7	9734	W	Serbia	D
Purcsin	1	9822	T, W	Hungary	C
Ruža Bijela	5	10419	W	Croatia	C
Seducha	1	10855	W	Serbia	C
Semillon	1	11480	W	France	D
Sremska Zelenika	3	15934	W	Balkan	C
Stanušina Crna	1	11994	W	North Macedonia	C
Tamjanika Crna	6	8057	T, W	Balkan	C
Villard Blanc	1	13081	W	France	C
Začinak	4	13400	W	Serbia	D
Žilavka	1	13446	W	Bosnia and Hercegovina	C
Zunić	1	15923	W	Balkan	A
Župljanka	1	13480	W	Serbia	D

a According to *VI*VC information.

b T, Table grape variety; W, Wine grape variety.

c SUN, Formerly USSR.

Most of the 49 identified genotypes were found to be varieties from the Western Balkans or neighbouring regions (**Table 1**). Our results indicate a clear dominance of two varieties: ‘Pamid’, and ‘Braghina Rosie’ (**Table 1**). Thus, we identified 29 samples as the red-berried Bulgarian variety ‘Pamid’, from plants usually prospected as “Plovdina” or “Lisičina” (**Supplementary Material 1**), which is the official name given to this variety in Serbia (Bešlić et al., 2012). Besides, we identified 15 samples as the Serbian variety ‘Braghina Rosie’. All samples identified as ‘Braghina Rosie’ were collected as “Bagrina”, the name used in Serbia to refer to this variety. In addition, other samples were identified as ancient table grape varieties from Eastern regions (like ‘Agadai’, ‘Chaouch Blanc’, and ‘Parmak Cerven’), traditional wine grape varieties from Western countries (like ‘Chasselas Blanc’, ‘Pinot Noir’, and ‘Semillon’), or varieties obtained in diverse breeding programs (like ‘Ferdinand de Lesseps’, ‘Villard Blanc’, and ‘Župljanka’) (**Table 1**).

The genetic analyses confirmed some previously known synonyms for several Balkan varieties (**Supplementary Material 1**). This is the case of ‘Bele Kozije Sise’, ‘Gavran’, ‘Muscat Krokan’, and ‘Smederevka’, to refer to the varieties ‘Coarna Alba’, ‘Grk Crni’, ‘Muscat Fleur D’Oranger’, and ‘Dimyat’, respectively. In addition, we found new potential local synonyms for other varieties. For example, five plants named “Tamjanika Ćilibarka” were successfully identified as ‘Muscat a Petits Grains’. Since these plants had white berries, the local name “Tamjanika Ćilibarka” can be considered as a new synonym of ‘Muscat a Petits Grains Blancs’. Interestingly, the term “tamjan” means “incense” in Serbian, so it is likely used to reflect the typical scent of Muscat varieties. In fact, this term is commonly included in the name of other Balkan varieties of the Muscat family, like ‘Tamjanika Crna’ and ‘Tamjanika Bela’ (Bešlić et al., 2012). In addition, “ćilibarka” derives from the Bosnian term “ćilibar”, which means “amber”, and it could be used to reflect the yellow-golden colour of ‘Muscat a Petits Grains Blancs’ mature berries. Similarly, we analysed four samples named “Jagoda”, which were identified as the interspecific hybrid variety ‘Ferdinand de Lesseps’. The term “jagoda” means “strawberry” in Serbian, and probably is used to reflect the strawberry-like and fruity aroma that characterizes “Jagoda” grapes and wines, also present in the *foxy* character of some *labrusca* varieties like ‘Ferdinand de Lesseps’ (Rogers and van Wyk, 2000). Therefore, the name “Jagoda” should be considered a new synonym of ‘Ferdinand de Lesseps’. Other potential new local synonyms identified in this work are “Ranka Bela” for ‘Ruža Bijela’, “Sura Lisičina” for ‘Blaufraenkisch’, “Osipač Crveni” for ‘Negru Virtus’, and “Ilijsko Rano” for ‘Csaba Gyongye’. Regarding the last one, the Serbian term “rano” means early, which might be used to evocate the early ripening trait that characterizes ‘Csaba Gyongye’ grapes (He et al., 2023).

### 3.2 Parentage analyses

**Figure 1:**
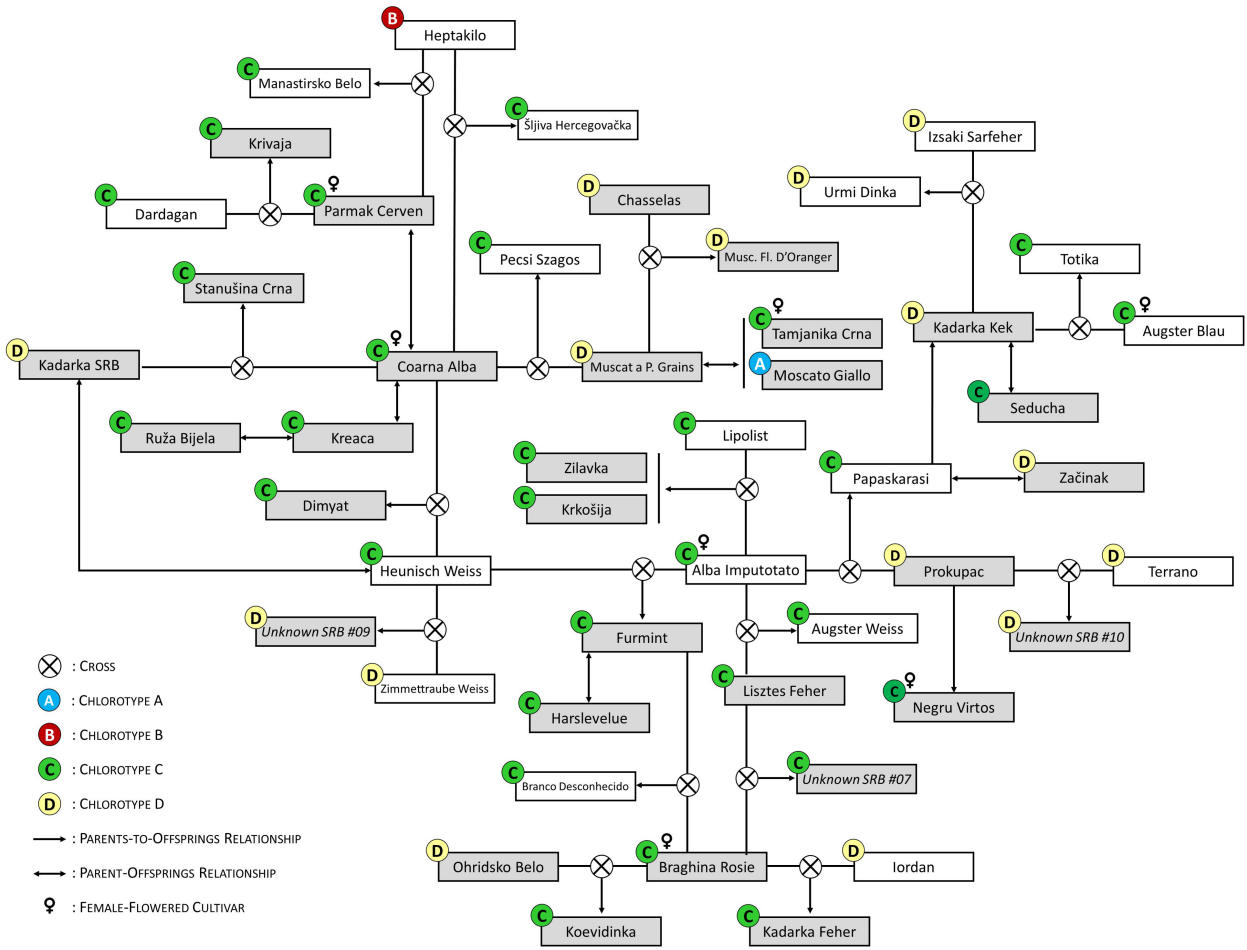
Selected parentage relationships between the grapevine genotypes identified after the prospection of 138 grapevines in old Serbian vineyards. Genotypes identified in this study are shown with a grey background colour. Different chlorotypes **(A, B, C or D)** are indicated in circles with different colours, according to the inset. For the full information of the parentage relationships identified in this work, see **Supplementary Materials 4** and **5**.

We merged the 59 non-redundant grapevine SNP profiles obtained in this work with those stored in the ICVV-DNA database for a wide search of possible first-order kinship relationships. Parentage analyses revealed 35 full pedigrees, including 17 of autochthonous varieties from the Balkans or other regions, 14 of varieties derived from different international or national breeding programs, and four of the unidentified genetic profiles obtained in this work (**Supplementary Material 4**). All detected pedigrees are highly reliable, given their high LOD values (> 50) and the null or low number of mismatching SNP and SSR alleles. In addition, we identified 29 duos (putative parent-offspring relationships) between two genotypes (**Supplementary Material 5**). As shown in **Figure 1**, our results indicate a leading role of ‘Alba Imputotato’, ‘Braghina Rosie’, and ‘Coarna Alba’ in the generation of Balkan autochthonous varieties, being involved as progenitors in 5, 4, and 4 full pedigrees, respectively. In addition, we identified five duos for ‘Coarna Alba’ (**Supplementary Material 5**). These three important progenitors have functionally female flowers, so they acted as mother in all their crosses. This feature contributed to the dissemination of their chloroplast type (chlorotype C for all of them) in the region. In fact, chlorotype C was the most abundantly found among the genotypes identified in this work (in 33 genotypes, 56.9%), followed by chlorotype D (18 genotypes, 31.0%), and chlorotype A (6 genotypes, 10.3%). Chlorotype B was only found once, in the alleged Moldavian variety ‘Coarna Neagra’ (**Table 1**). Apart from these three major founders, we found several descendants of ‘Chasselas’, ‘Heunisch Weiss’, and ‘Kadarka Kek’, although some of them are obtentions derived from national or international grape breeding programs, like ‘Jo Rizling’ (‘Chasselas’ × ‘Welschriesling’), ‘Knipperle’ (‘Pinot’ × ‘Heunisch Weiss’), and ‘Probus’ (‘Kadarka Kek’ × ‘Cabernet Sauvignon’) (**Supplementary Materials 4**, **5**).

Among other findings, we identified for the first time the full pedigree of the varieties ‘Koevidinka’ (‘Braghina Rosie’ × ‘Ohridsko Belo’), ‘Krivaja’ (‘Parmak Cerven’ × ‘Dardagan’), ‘Manastirsko Belo’ (‘Parmak Cerven’ × ‘Heptakilo’), ‘Stanušina Crna’ (‘Coarna Alba’ × ‘Kadarka SRB’), and ‘Šljiva Hercegovačka’ (‘Coarna Alba’ × ‘Heptakilo’) (**Figure 1**; **Supplementary Material 4**). Likewise, we identified the full pedigree of the unidentified genetic profiles “*Unknown SRB 03*” (‘Alicante Henri Bouschet’ × ‘Gamay Noir’), “*Unknown SRB 07*” (‘Braghina Rosie’ × ‘Lisztes Feher’), “*Unknown SRB 09*” (‘Zimmettraube Weiss’ × ‘Heunisch Weiss’), and “*Unknown SRB 10*” (‘Terrano’ × ‘Prokupac’) (**Figure 1**; **Supplementary Material 4**). We also found a duo between “*Unknown SRB 05*” and the Bulgarian variety ‘Berbecel’ (**Supplementary Material 5**). Our results were also useful to complete the pedigree information of the Serbian variety ‘Krkošija’ (‘Alba Imputotato’ × ‘Lipolist’), for whom only one progenitor had been indicated before (www.vivc.de). In addition, we could provide strong evidence to correct some pedigrees indicated in the literature, like those of ‘Branco Desconhecido’, ‘Ferdinand de Lesseps’, ‘Kadarka Feher’, and ‘Pecsi Aldas’.

### 3.3 Relationship between Balkan varieties and varieties from other regions

The unrooted dendrogram calculated to explore the genetic relationships between 43 local genotypes revealed seven different clusters (**Supplementary Material 6**). This clustering mostly reflected the main family networks observed in the pedigree analysis. For example, group 1 clustered ten varieties, many of them genetically related to ‘Alba Imputotato’, group 2 clustered ‘Coarna Alba’ and five of its first- or second-degree descendants, group 3 ‘Braghina Rosie’ (syn. ‘Bagrina’) and some of its descendants, group 5 clustered three varieties with a parent-offspring relationship with ‘Vulpea’, and group 6 some varieties related to ‘Heunisch Weiss’. In addition, group 7 clustered 6 genotypes, including two Muscat varieties (‘Muscat a Petits Grains’ and ‘Tamjanika Crna’), and group 4 clustered two varieties with no apparent parentage relationship (‘Coarna Neagra’, and ‘Mustoasa de Maderat’).

**Figure 2:**
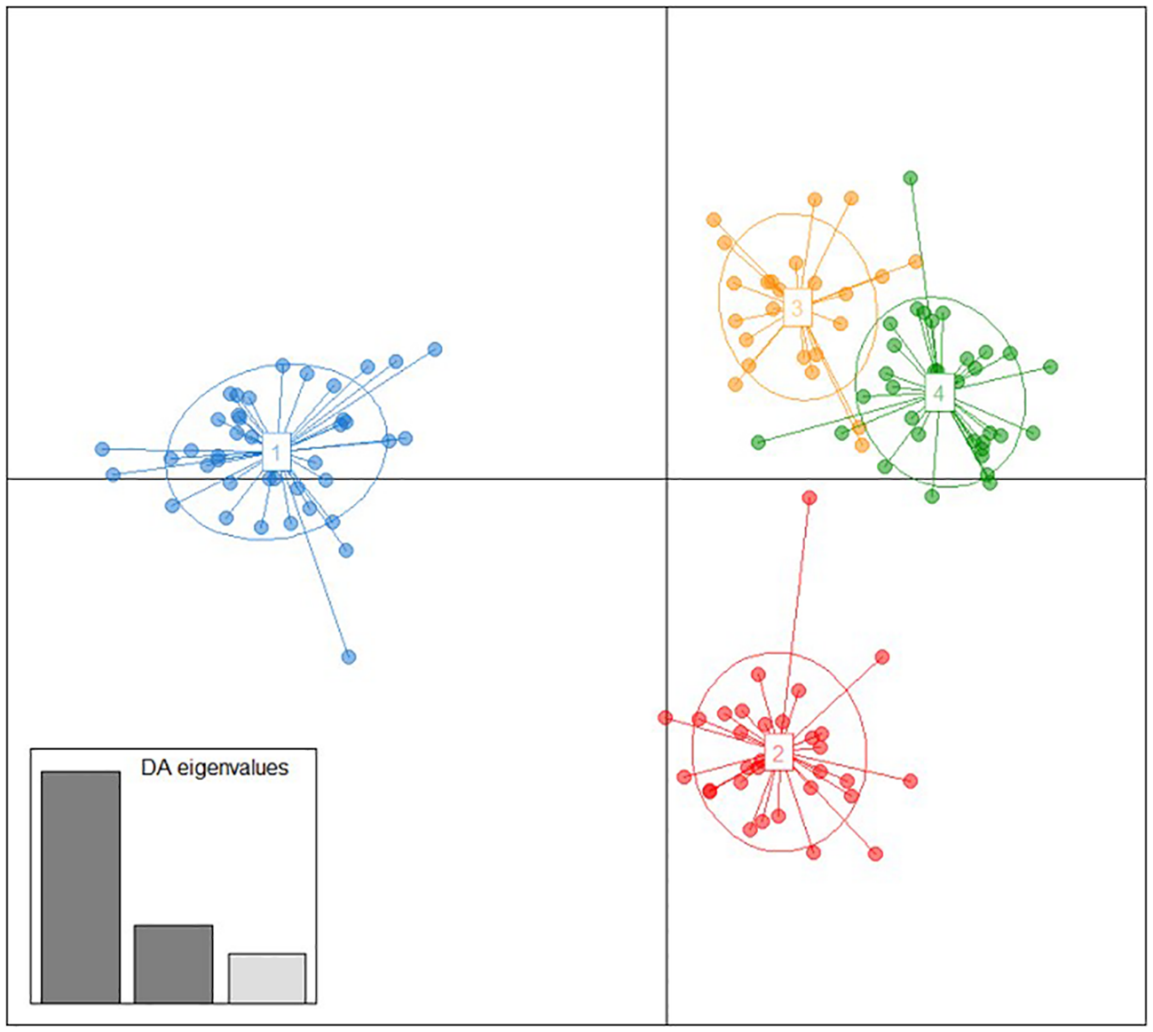
Discriminant analysis of principal components (DAPC) obtained between the Balkan grapevine varieties identified in this work and some selected varieties of the Caucasus, Central Europe, and the Iberian Peninsula genetic pools. Grapevine varieties are shown as dots, and the four identified groups as inertia ellipses, with colours denoting group allocation (group 1: blue; group 2: red; group 3: orange; group 4: green), detailed in the **Supplementary Materials 2** and **3**. Eigenvalues of the discriminant analysis are graphically displayed in the inset.

The genetic relationship of these local genotypes with some from three other geographic regions (Caucasus, Iberian Peninsula and Central Europe) was firstly explored by means of a DAPC analysis. As expected, this analysis revealed four clusters of genetic similarity (**Figure 2**), which represented the four origins of the varieties used for the analysis: group 1 clustered 42 varieties from the Balkans; group 2 clustered 30 varieties from the Caucasus and one from Central Europe (‘Aramon Noir’); group 3 clustered 22 varieties from the Iberian Peninsula and three from Central Europe (‘Carignan’, ‘Danugue’, and ‘Olivette Blanche’); and group 4 clustered 26 varieties from Central Europe, eight from the Iberian Peninsula (‘Alfrocheiro’, ‘Alvarelhao’. ‘Alvarinho’, ‘Borracal’, ‘Loureiro Blanco’, ‘Maturana Blanca’, ‘Molar’, and ‘Touriga Nacional’), and one unidentified genotype from the Balkans (“*Unknown SRB 06*”) (**Supplementary Materials 2**, **3**, **7**). These four groups were well separated in the scatterplot, but for some overlapping between groups 3 and 4, which represented Iberian and Central Europe varieties, respectively. Regarding the Balkan varieties, this analysis revealed a clear genetic differentiation from the other geographic backgrounds included in the analysis.

**Figure 3:**
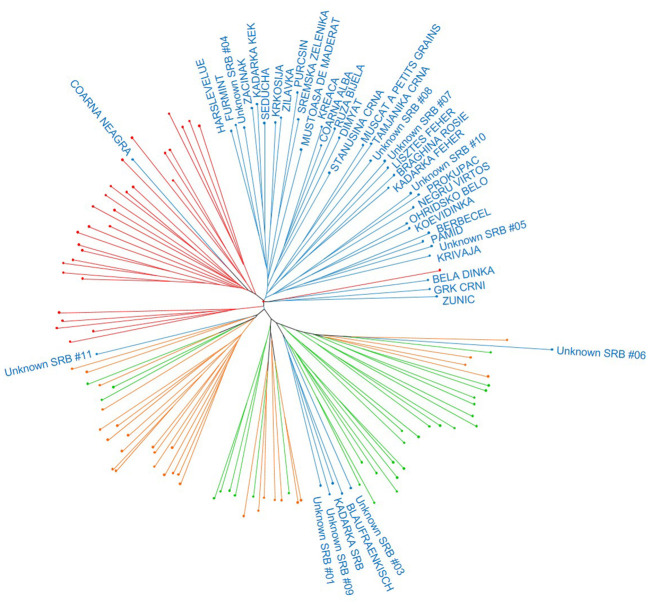
Unweighted Neighbor-Joining (UwNJ) radiation tree obtained between the Balkan grapevine varieties identified in this work and some selected varieties of the Caucasus, Central Europe, and the Iberian Peninsula genetic pools. Grapevine varieties are shown as dots, whose colour indicate its region of origin according to the *V*IVC database (Balkans: blue; Caucasus: red; Iberian Peninsula: orange; Central Europe: green). For the sake of clarity, only the name of the Balkan varieties are included. The complete list of varieties is listed in the **Supplementary Material 2**.

This general clustering was supported by the results obtained in the dendrogram, in which most of the varieties from the Balkans were clearly separated in one independent branch (**Figure 3**). Likewise, most of the Caucasian samples clustered in one single branch, whilst Iberian and Central Europe varieties were distributed in other three major branches. Interestingly for this study, five Balkan genotypes (‘Blaufraenkisch’, ‘Kadarka SRB’, “*Unknown SRB 01*”, “*Unknown SRB 03*”, and “*Unknown SRB 09*”) were found to localize with some Central Europe varieties, many of them genetically related to the variety ‘Heunisch Weiss’. On the other hand, ‘Coarna Neagra’ was found to cluster with most of the samples from the Caucasus, and “*Unknown SRB 06*” and “*Unknown SRB 11*” were found to cluster in different branches of the dendrogram, next to some Iberian and Central European varieties.

## 4 Discussion

### 4.1 Historical events shaped current Serbian varietal assortment

The study of the genetic resources from traditional winemaking regions helps to understand the evolution of local viticulture systems. The Western Balkans is considered a main crossroad between Western and Eastern European civilizations (Cvijić, 1918). This strategic location prompted the exchange of multiple commodities between distant regions for centuries, including grapevine varieties. During their history, territories of current Western Balkans countries shared not only a common border, but also many traditions and agricultural practices. It included sharing some viticulture practices, like the common use of distinctive grapevine varieties. As a result, studies that nowadays explore the grapevine genetic resources of one specific Western Balkan country or Balkan region usually identify varieties attributed to close territories now constituting adjacent nations, where they are grown under local synonyms (Maletic et al., 1999; Štajner et al., 2015; Žulj Mihaljević et al., 2015; Carka et al., 2015; Maraš et al., 2020). Accordingly, we did not only identify putative autochthonous varieties from Serbia, but also from other Western Balkans countries (**Table 1**).

Our analyses are not aimed to reveal for how long the varieties identified in this work have been cultivated in the country. In fact, this objective will need of a direct comparison between archaeological and modern samples to provide a historical frame (Ramos-Madrigal et al., 2019; Cohen et al., 2023), which, unfortunately, is not feasible in most cases. However, varieties like ‘Plovdina’ (syn. ‘Pamid’), ‘Prokupac’, ‘Smederevka’ (syn. ‘Dimyat’), and ‘Začinak’ are considered to have been grown in Serbia for centuries (Bešlić et al., 2012). For example, the variety ‘Smederevka’ (syn. ‘Dimyat’) is suggested to have been cultivated in the Serbian region of Smederevo since the 3^rd^ century BCE (Dabić Zagorac et al., 2019). Genetic analyses revealed that this variety is the offspring between ‘Coarna Alba’ and ‘Heunisch Weiss’ (Lacombe et al., 2013), two grapevine varieties of ancient origin that acted as founders of multiple Balkan grapevines (Lacombe et al., 2013; Maul et al., 2015; Maraš et al., 2020). ‘Prokupac’ is also meant to be an ancient Serbian variety, which is now cultivated in all Serbian winegrowing regions (Bešlić et al., 2012). Here, we found that ‘Prokupac’ is the founder of a family lineage of up to four continuous generations of varieties related to the Balkans (**Figure 1**), supporting its presence in the region for a long time.

The Ottoman period (14^th^ to 19^th^ centuries) expanded table grape production across the Balkans, which promoted the cultivation of Eastern varieties like ‘Afuz Ali’ (syn. ‘Afus Ali’), Čauš (syn. ‘Chaouch Blanc’), ‘Drenak’ (syn. ‘Parmak Cerven’), and ‘Sultana’ (syn. ‘Sultanina’) (Dabić Zagorac et al., 2019). The characterization of marginal germplasm found in traditional winemaking regions of different Balkan countries has been useful to support the old presence of some of these Eastern table grape varieties in the Western Balkans, like the Turkish varieties ‘Chaouch Blanc’ and ‘Parmak Cerven’ (Carka et al., 2015; Maraš et al., 2020). In fact, some of them have been found to be key progenitors in the generation of a high number of current Balkan varieties, supporting their long-standing cultivation in the region and their impact on regional varietal assortment (Carka et al., 2015; Maraš et al., 2020). Accordingly, here we identified some old Serbian grapevines as the Eastern cultivars ‘Agadai’, ‘Chaouch Blanc’, and ‘Parmak Cerven’ (**Table 1**), and we identified that some of them were involved in the origin of alleged Serbian varieties, like ‘Krivaja’ (‘Parmak Cerven’ × ‘Dardagan’). These findings agree with previous reports, and support how the table grapes varieties introduced during the Ottoman period influenced current grapevine Balkan germplasm.

During the Ottoman period, grapes cultivated for winemaking still played an important role in the economic development and social life of Serbia. In fact, reports of that time indicate the name of many grapevine varieties grown in vineyards of the Fruška Gora winemaking region (in North Serbia), including “Beli Grašac”, “Crni Grašac”, “Čavčica”, ‘Skadarka’ (syn. ‘Kadarka Kek’), ‘Slankamenka’ (syn. ‘Slankamenka Bela’), and ‘Tamjanika Crna’ (Bolić, 1816). In the post-Ottoman era, the interest for recovering the winemaking industry led to the establishment of new vineyards all over the country. Some reports indicate the relevance of varieties like ‘Skadarka’ (syn. ‘Kadarka Kek’), ‘Tamjanika Crna’, and “Čađavica”, which dominated in relevant winemaking regions of Srem (Syrmia) and Fruška Gora (Lambl, 1873; Fotić, 2020). Some of these varietal names are recognized today as grape varieties grown in Serbia for winemaking, like ‘Kadarka Kek’, ‘Slankamenka Bela’, ‘Tamjanika Crna’ and “Beli Grašac” (“Grašac” is the local synonym used in Vojvodina (northern Serbia) for the white-berried cultivar ‘Welschriesling’), and some of them were identified in our work, suggesting their continuous cultivation in the country for centuries. However, to our knowledge, names like “Čađavica”, “Čavčica”, and “Crni Grašac” are not recognized nowadays as grapevine varieties. It might be indicating that these varieties are cultivated under alternative names, or that they might have disappeared under different pressures, like the phylloxera crisis occurred at the end of the 19^th^ century. In fact, the arrival of this pest in 1882 affected the flourishing post-Ottoman situation of the Serbian sector by devastating many vineyards of own-rooted grapevines, which were frequently replaced by grafted vines or by own-rooted hybrid varieties (Bešlić et al., 2012). In 1889, associations of grape growers and winemakers met at the “First national public meeting of winemakers in Serbia”, where they promoted the propagation and planting of ‘Začinak’, ‘Skadarka’ (syn. ‘Kadarka Kek’), ‘Kameničarka’ (syn. ‘Prokupac’), ‘Smederevka’ (syn. ‘Dimyat’), and ‘Dinjka’ (syn. ‘Brugnara’) as a way to boost the obtaining of red and white wines from local varieties (Maksimović et al., 2021). In addition, other varieties of western origin like ‘Traminac’ (syn. ‘Traminer’/’Savagnin’), ‘Pinot Noir’, ‘Pinot Blanc’, and ‘Gamay’ were introduced in the country for the first time. Likely reflecting these western introductions, we identified some old plants as the French varieties ‘Pinot Noir’, and ‘Semillon’ (**Table 1**). Similarly, the identification in this work of some old plants as the hybrids ‘Ferdinand de Lesseps’ and ‘Villard Blanc’ could reflect the historical massive incorporation of hybrid varieties occurred in Europe as a solution against phylloxera and other pests (Wagner, 1955), also observed in other Balkan countries (Maraš et al., 2020). Due to their hybrid origin, these varieties hold some level of pest resistance, so they were planted on a considerable scale for the direct production of wine (Töpfer et al., 2011). However, the low wine quality and some legal regulations led to their gradual disappearance (Wagner, 1955).

In the last decades, grapevine varietal diversity in Serbia expanded considerably by the incorporation of new wine grape varieties, in most cases new breds obtained from the hybridization of local varieties (Nikolic et al., 2015). In this line, new varieties like ‘Godominka’, ‘Probus’, and ‘Župski Bojadiser’ have been successfully incorporated by local winemakers to diversify the production of white and red wines. Consistent with this trend, we detected a sample of ‘Župljanka’, a white-berried variety created in 1970 at the Serbian Institute of Viticulture and Fruit Growing in Sremski Karlovci (‘Prokupac’ × ‘Pinot Noir’).

### 4.2 Serbian varieties are part of a complex pedigree network integrating germplasm from different Balkan regions

Genetic diversity analyses in different viticulture areas have detected that regional grapevine germplasm can be linked to specific founders, like ‘Cabernet Franc’ in Aquitaine (France) (Boursiquot et al., 2009), ‘Hebén’ in the Iberian Peninsula (Zinelabidine et al., 2015), or ‘Kratošija’ (syn. ‘Primitivo’) and ‘Parmak Cerven’ in Montenegro (Maraš et al., 2020). Here, we found that three female varieties (‘Alba Imputotato’, ‘Braghina Rosie’, and ‘Coarna Alba’) played a major role in the generation of the grapevine germplasm of the Balkans (**Figure 1**). The relevance of female varieties to generate local diversity has been frequently observed in other winemaking regions (Zinelabidine et al., 2015; Cunha et al., 2020; Raimondi et al., 2020). Female varieties have flowers with sterile pollen grains, so they need to outcross to generate viable seeds. This process generally results in vigorous and high-yielding plants, as it overcomes the negative impacts of inbreeding depression on plant fertility and reproductive performance shown by the progeny of selfed hermaphrodite varieties (Cattonaro et al., 2014). As evidenced from the low number of varieties known to derive from selfing events (Cipriani et al., 2010; Maraš et al., 2020), the empirical observation that seedlings from female varieties were more vigorous and productive likely favoured that early farmers used the seeds of those varieties for grapevine propagation (Cattonaro et al., 2014), when the use of cuttings was not feasible. By doing so, they were generating new genotypes with beneficial features that were the base of new varieties. In this line, female varieties need to be pollinated by nearby pollen donors (generally hermaphrodite varieties) for regular grape production. This situation ultimately derives on the identification of multiple full-siblings in a given specific region. These are the cases of ‘Castelao Branco’, ‘Malvasia Fina’, and ‘Trincadeira das Pratas’ (offspring of crosses between ‘Hebén’ and ‘Alfrocheiro’), ‘Viura’ and ‘Xarello’ (‘Hebén’ × ‘Brustiano Faux’), ‘Crna Tomba’ and ‘Raceska’ (‘Parmak Cerven’ × ‘Primitivo’), ‘Brachetto Migliardi’ and ‘Malvasia di Casorzo’ (‘Malvasia Aromatica di Parma’ (syn. ‘Malvasia Casalini’) × ‘Lambrusca di Alessandria’) (Cunha et al., 2015; Zinelabidine et al., 2015; Maraš et al., 2020; Raimondi et al., 2020), and ‘Krkošija’ and ‘Žilavka’ (‘Alba Imputotato’ × ‘Lipolist’), two full-siblings identified in this work.

We identified an intricate parentage network linking multiple grapevine varieties not only from Serbia, but from other Balkan countries (**Figure 1**), which results from an intense exchange of plant material among Balkan regions for centuries (Štajner et al., 2014; Maraš et al., 2020). Within this network, we identified the full pedigree of some local varieties, not previously reported in the literature (like ‘Koevidinka’, ‘Krivaja’, ‘Manastirsko Belo’, ‘Stanušina Crna’, and ‘Šljiva Hercegovačka’), which contributes to the knowledge of Balkan varieties. Likewise, we confirmed the suggested pedigrees of ‘Probus’ and ‘Radmilovački Muskat’, two grape varieties bred in Serbia at the end of the 20^th^ century. We also identified the full pedigree of four unidentified varieties: “*Unknown SRB #03*”, “*Unknown SRB #07*”, “*Unknown SRB #09*”, and “*Unknown SRB #10*”. “*Unknown SRB #03*” was found only once across the samples studied in this work, but the pedigree identified matches with that of the varieties ‘Tinturier Youpski’ and ‘Župski Bojadiser’ (*V*IVC varieties number 12517 and 13481, respectively). The term “bojadiser” means teinturier in Serbian, and “Župski Teinturier” is used as a synonym of ‘Župski Bojadiser’. Thus, ‘Tinturier Youpski’ is very likely a synonym of ‘Župski Bojadiser’, a teinturier variety bred in 1979 at the University of Belgrade (Milišić et al., 2021). However, the sample “*Unknown SRB #03*” does not correspond to a specimen of ‘Župski Bojadiser’, since it does not have red-fleshed berries. Thus, this genotype is a full-sibling of ‘Župski Bojadiser’, probably obtained at the same breeding cross. On the other hand, “*Unknown SRB #07*”, “*Unknown SRB #09*”, and “*Unknown SRB #10*” were also found once across the samples analysed in this work, but their genetic profiles matched those of three samples previously analysed in our laboratory (**Supplementary Material 1**), suggesting that they are true varieties. Interestingly, “*Unknown SRB #10*” was found to be the offspring between ‘Terrano’ and ‘Prokupac’. This pedigree matches with that of the Serbian variety ‘Rumenika’ (*V*IVC variety number 15481), a black-berried variety obtained at the Faculty of Agriculture in Sremski Karlovci (Serbia) in 1982-1983 (Cvejic et al., 2016). Nowadays, ‘Rumenika’ is commonly grown in the Vojvodina region, where the sample with the genetic profile “*Unknown SRB #10*” was collected. Thus, “*Unknown SRB #10*” could correspond to the grapevine variety ‘Rumenika’, or they might be full-siblings. Unfortunately, we could not find a reference profile for ‘Rumenika’ for a direct comparison with the genetic profile of “*Unknown SRB #10*”.

Parentage results were also useful to refute some previously reported pedigrees. The interspecific variety ‘Ferdinand de Lesseps’ (which corresponds to the variety known in Serbia as “Jagoda”) is thought to have originated from the cross of ‘Chasselas Blanc’ and ‘Isabella’. However, their genetic profiles do not support this pedigree, since ‘Ferdinand de Lesseps’ has many SSR alleles that are not present in ‘Chasselas Blanc’ or ‘Isabella’ (VVS2^149^, VVMD5^234^, VVMD28^244^, VrZAG62^186^, VrZAG79^255^, www.vivc.de). Our data did not provide any reliable full pedigree for ‘Ferdinand de Lesseps’, but they were useful to find a compatible parent-offspring connection between this variety and ‘Muscat Hamburg’ (**Supplementary Material 4**). Given that the pedigree of ‘Muscat Hamburg’ (‘Schiava Grossa’ × ‘Muscat of Alexandria’) is fully supported by molecular analyses (Crespan, 2003), our results indicate that ‘Muscat Hamburg’ could be one of the parents of ‘Ferdinand de Lesseps’. This direction is plausible, since ‘Muscat Hamburg’ was widely used by grape breeders to obtain hybrids adapted to cool-climate conditions (like ‘Downing’ ‘Mills’, or ‘Quassaic’) before ‘Ferdinand de Lesseps’ was exhibited in 1870 (Hedrick, 1908) (Rogers and van Wyk, 2000). Interestingly, early descriptions of ‘Ferdinand de Lesseps’ grapes consider the taste of ‘Ferdinand de Lesseps’ as “a mixture of muscat and strawberry” (du Plessis, 1925), suggesting the involvement of a Muscat variety in its pedigree.

Similarly, the variety ‘Kadarka Feher’ is reported as a cross between ‘Bakator Belyi’ and ‘Kadarka’. “Kadarka” is used as a synonym of two varieties: ‘Olasz Kadarka’ and ‘Kadarka Kek’. However, ‘Kadarka Feher’ has multiple SSR alleles that are not present in ‘Bakator Belyi’ or ‘Olasz Kadarka’ (VVS2^135^, VVMD5^242^, VVMD27^180^, VVMD28^236^, VVMD28^260^, www.vivc.de). Similarly, it has many alleles not present in ‘Bakator Belyi’ or ‘Kadarka Kek’ (VVMD5^242^, VVMD27^180^, VVMD28^236^, VrZAG62^196^, www.vivc.de). So, ‘Kadarka Feher’ did not originate from any of these two combinations. In this regard, our results confidently support that ‘Kadarka Feher’ resulted from the cross between ‘Braghina Rosie’ and ‘Iordan’. Likewise, the variety ‘Pecsi Aldas’ is suggested to be an interspecific cross of ‘Bicane’ and ‘Golden Queen’. However, ‘Golden Queen’ is homozygous for some alleles (VVS2^151^, VVMD7^249^, VrZAG79^247^, www.vivc.de) that are not present in ‘Pecsi Aldas’, which is heterozygous for those microsatellite markers. In this regard, our results indicate that ‘Pecsi Aldas’ is not an interspecific hybrid, and it resulted from the cross between the *V. vinifera* L. varieties ‘Bicane’ and ‘Chasselas’. Lastly, ‘Branco Desconhecido’ was previously suggested as an offspring of the varieties ‘Furmint’ and ‘Kadarka Bela’ (Cunha et al., 2020). However, our results confidently support that it derived from the cross between ‘Braghina Rosie’ and ‘Furmint’.

### 4.3 Many Balkan varieties could descend from the ancient variety ‘Visparola’

Traditional and molecular-driven classification systems divide *V. vinifera* L. varieties into three major genetic groups (Negrul, 1946; Bacilieri et al., 2013; Laucou et al., 2018). More recently, and based on whole genome sequencing data, up to six ancestral genetic groups have been identified (Dong et al., 2023). All these different approaches agree in the identification of one genetic group mostly formed by Balkan wine grape varieties, which highlights the distinctiveness of this genetic pool. Our results also support this singularity, as the Balkan genotypes identified in this work markedly differed from the three different genetic pools used for comparative purposes (**Figures 2**, **3**). Varieties from these ancestral groups are also known to have important phenotypic differences, including traits related to phenology, yield components, and oenological potential (Houel et al., 2013; Nicolas et al., 2016; Migicovsky et al., 2017; Sikuten et al., 2021). Many studies highlight the phenotypic singularity of several Balkan wine grape varieties compared to those of international relevance (Pajović-Šćepanović et al., 2018; Banjanin et al., 2024), and some of them have been even used to breed new varieties with great oenological potential worldwide (Cvejic et al., 2016). Altogether, these works point out the possibilities of Balkan varieties as a novel source of innovation for the winemaking industry, and remark the interest of unravelling the evolutionary mechanisms that lead to their differentiation.

**Figure 4:**
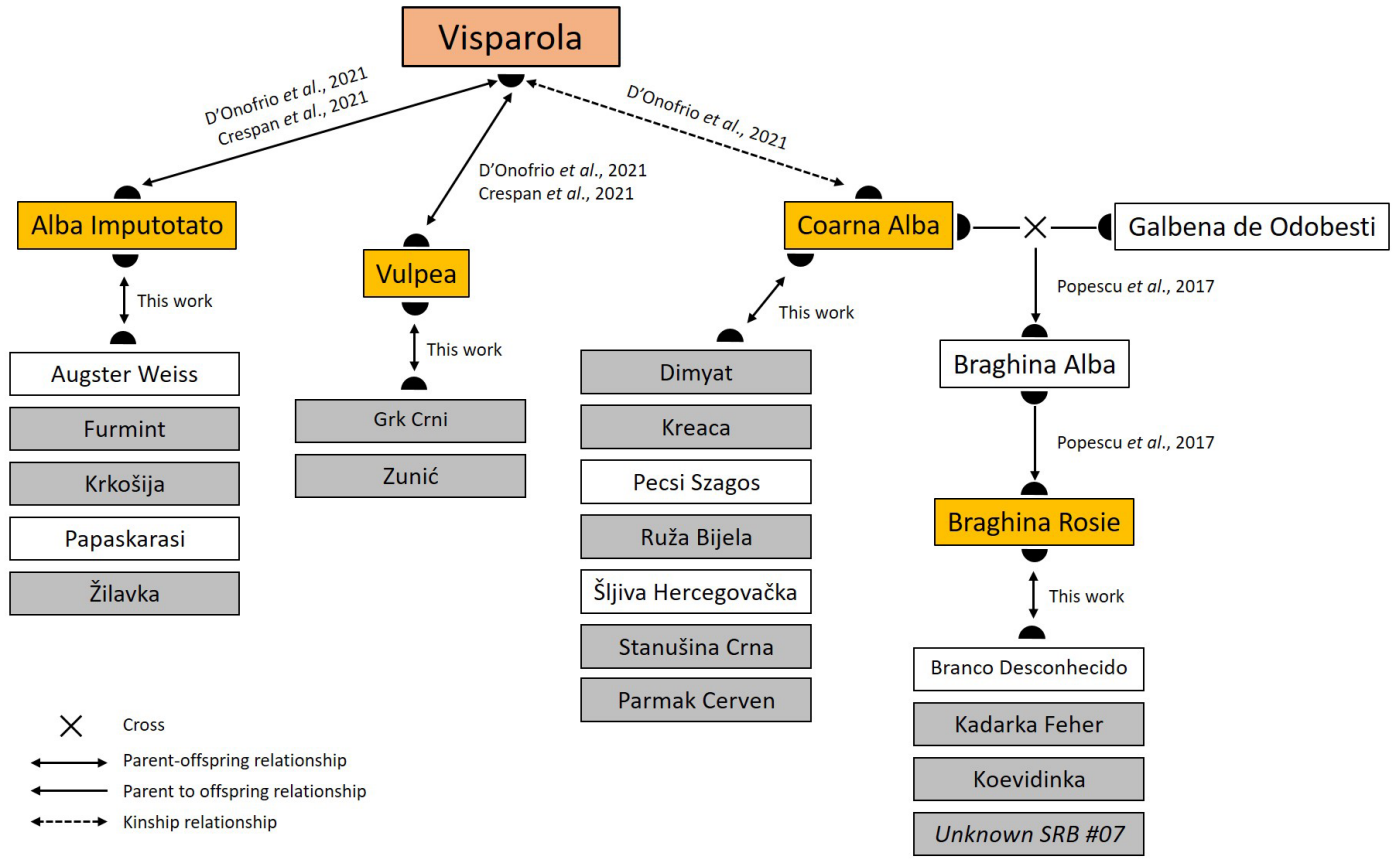
Genetic relationships linking four of the major founders of Balkan genotypes identified in this work with the ancient variety ‘Visparola’. Major local founders are shown in yellow, whilst genotypes found in Balkan vineyards related to them are shown in grey. For simplicity, only those with a direct parent-offspring relationship are shown. For the full list and information of the parentage relationships identified in this work, see Online Resources 4 and 5.

Although our study is based on a reduced number of Serbian varieties, the cluster analysis clearly subdivided them into a series of groups of distinct genetic backgrounds (**Supplementary Material 6**). Interestingly, these groups reflect some family relationships that could be easily traced back to a reduced set of founders: ‘Alba Imputotato’ (group 1), ‘Braghina Rosie’ (3), ‘Coarna Alba’ (2), ‘Heunisch Weiss’ (6), and ‘Vulpea’ (5). Interestingly, both ‘Alba Imputotato’ and ‘Vulpea’ have a direct parent-offspring with ‘Visparola’ (Crespan et al., 2021; D’Onofrio et al., 2021), an ancient variety that is also genetically connected with ‘Coarna Alba’ (D’Onofrio et al., 2021). Based on 13 SSRs, Popescu et al. (2017) suggested that ‘Braghina Rosie’ has a first-degree relationship with the alleged Romanian variety ‘Braghina Alba’, which is meant to be the offspring of ‘Coarna Alba’ × ‘Galbena de Odobesti’. Thus, ‘Braghina Rosie’ could be the son and grandson of ‘Braghina Alba’ and ‘Coarna Alba’, respectively, and therefore also genetically linked to ‘Visparola’. Consequently, we can consider that ‘Alba Imputotato’, ‘Braghina Rosie’, ‘Coarna Alba’, and ‘Vulpea’ acted as important direct local founders, whilst ‘Visparola’ was a major actor on the foundation of current Balkan germplasm from an upper position in their genealogical tree (**Figure 4**). The area of origin of ‘Visparola’ remains unknown; however, its inferred full sibship with the Greek variety ‘Avgoustiatis’ has served to suggest that ‘Visparola’ could have originated in the Southern Balkans (D’Onofrio et al., 2021). This Balkan origin is also supported by the numerous varieties found across the Balkans that are ultimately linked to ‘Visparola’, as reported here and in previous works (Štajner et al., 2015; Žulj-Mihaljevic et al., 2020; D’Onofrio et al., 2021). Interestingly, we found up to four continuous generations of the same family lineage starting from ‘Alba Imputotato’ and ‘Prokupac’, identifying two alleged Hungarian varieties (‘Urmi Dinka’ and ‘Totika’) as the great-great-grandsons of ‘Visparola’ (**Figure 1**). This agrees with the suggested origin (or a very ancient presence) of this variety in the Balkans (D’Onofrio et al., 2021). ‘Visparola’ has been recently appointed as a key ancestor of numerous varieties of Southern and Central Italy, and as a “bridge variety” linking Balkan and Italian grapevine genetic resources (D’Onofrio et al., 2021), probably through *Magna Graecia*, region of South Italy formerly populated by Greek colonizers where descendants of ‘Visparola’ have been found too (De Lorenzis et al., 2019). Our results reinforce the role of this variety in the Balkans, as well as an ultimate common origin of many current Balkan and Italian varieties through ‘Visparola’. The outstanding genetic legacy of this variety in the Balkans and the Italian Peninsula might partly explain why some genetic resources from both geographic regions tended to cluster together in different wide genetic structure and phylogenetic analyses (Emanuelli et al., 2013; Laucou et al., 2018).

Lastly, cluster analyses detected an additional independent contribution to the diversity of the Balkans through the role of the ancient, ubiquitous, and prolific variety ‘Heunisch Weiss’. This variety is known to be the origin of many renowned varieties from Central Europe like ‘Chardonnay Blanc’, ‘Gamay Noir’, and ‘Riesling Weiss’, and of some varieties grown at a commercial scale in the Balkans like ‘Iordan’ and ‘Francuse’ in Romania, and ‘Xynomavro’ in Greece (Maul et al., 2015). In addition, this variety has also been acknowledged as the progenitor of many underused varieties, many of them maintained in ampelographic collections (Lacombe et al., 2013; Štajner et al., 2015; Carka et al., 2015; Popescu et al., 2017). Our results remark the role of ‘Heunisch Weiss’ in the generation of Balkan varieties, through the detection of some additional genetic relationships to those previously described. Thus, ‘Kadarka SRB’ and one unidentified variety (“*Unknown SRB 09*”) were found to be parent-offspring related to ‘Heunisch Weiss’, so they become part of one of the most abundant families of grapevine varieties described so far. However, the prolificacy of ‘Heunisch Weiss’ led to the presence of its descendants in very divergent regions (ranging from Central Europe to the Southern Caucasus), which hinders setting its true place of origin (Maul et al., 2015). In any case, considering that some of its descendants produce some of the finest and most valued wines worldwide (Maul et al., 2015), it is worthy to evaluate how the novel ‘Heunisch Weiss’-derived varieties detected in this work would perform at an oenological level, to see if they could outperform some of its renowned western siblings.

## 5 Conclusions

As exemplified in this work, old vineyards from traditional winemaking regions have retained a high diversity of varieties despite a globalizing wine market. Now, these underused varieties have the potential to revitalize regional, national and international winemaking sectors, by providing “novel” alternatives to wine producers and consumers. These minor varieties may also have unique traits that might help solving some of the most pressing current viticulture challenges. So, they have to be collected and preserved to avoid their loss, and studied to discover these potential useful traits and evaluate their oenological potential. The analysis of the varieties identified in this work allowed linking their origin to some major Serbian historical events, which affected its varietal assortment by the disappearance and introduction of grapevine varieties over time. The analysis of their parentage relationships was useful to pinpoint a reduced number of varieties with a prominent role in the generation of local genetic resources. Interestingly, many of these founders could be linked to the ancient variety ‘Visparola’, whose genetic legacy might have imprinted a great impact on Balkan grapevine germplasm.

## Data availability statement

The original contributions presented in the study are included in the article/**Supplementary Material**. Further inquiries can be directed to the corresponding authors.

## Author contributions

JT: Conceptualization, Data curation, Formal analysis, Investigation, Methodology, Visualization, Writing – original draft, Writing – review & editing. ST: Data curation, Resources, Writing – review & editing. YF: Investigation, Writing – review & editing. MN: Data curation, Resources, Writing – review & editing. AS: Investigation, Writing – review & editing. DI: Resources, Writing – review & editing. ŽT: Conceptualization, Funding acquisition, Project administration, Resources, Writing – review & editing. MG: Conceptualization, Writing – review & editing. JM-Z: Conceptualization, Writing – review & editing. JI: Conceptualization, Data curation, Formal analysis, Funding acquisition, Investigation, Methodology, Project administration, Resources, Supervision, Writing – review & editing.
