# Supplementary material for: The genetic characterization of grapevines prospected in old Serbian vineyards reveals multiple relationships between traditional varieties of the Balkans
Source: Front Plant Sci. 2024 Jul 11;15:1391679. doi: 10.3389/fpls.2024.1391679 (PMC11269227; doi:10.3389/fpls.2024.1391679)
Supplement: Supplementary file 1 [file Image_1.pdf]

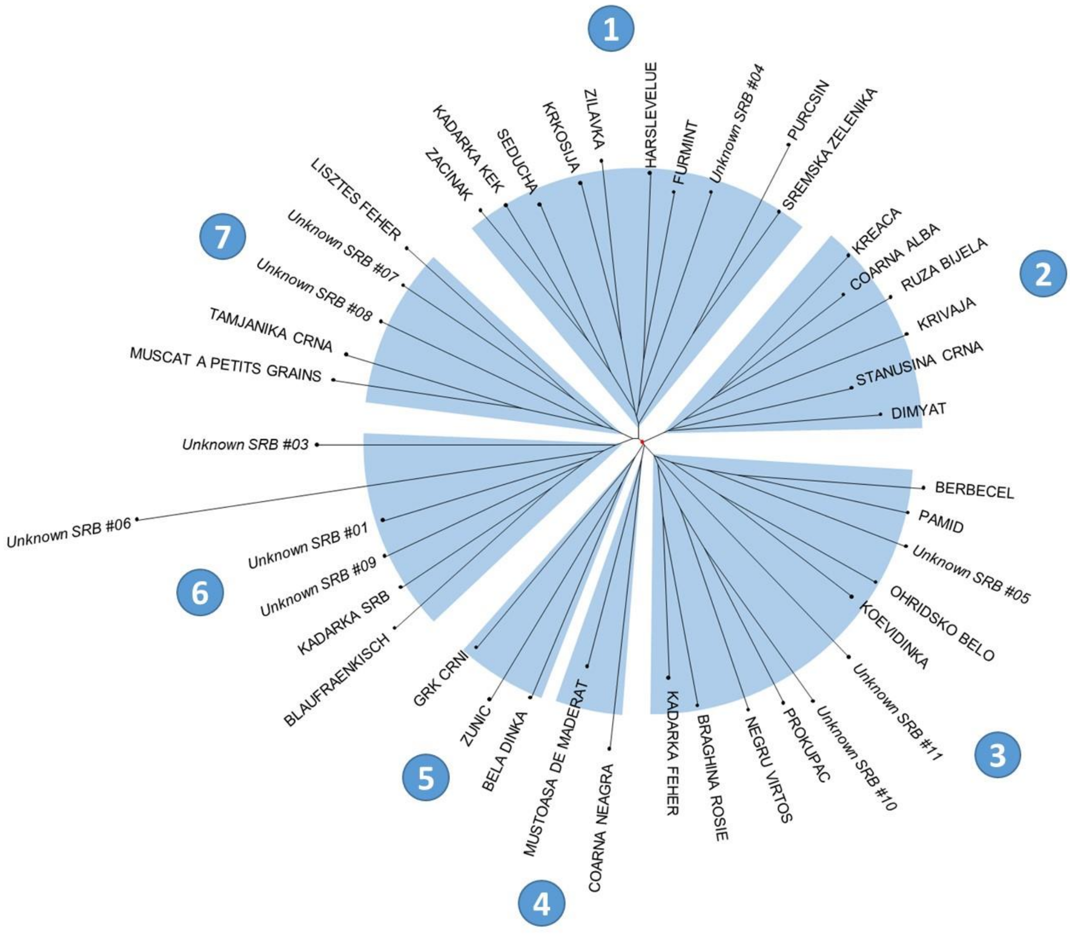

Supplementary Material 6. Unweighted Neighbor-Joining (UwNJ) radiation tree obtained between the 43 Balkan varieties identified in this work, based on 240-SNPs. All varieties were divided into seven genetic groups (1-7), indicated in blue.
