# Supplementary material for: The genetic characterization of grapevines prospected in old Serbian vineyards reveals multiple relationships between traditional varieties of the Balkans
Source: Front Plant Sci. 2024 Jul 11;15:1391679. doi: 10.3389/fpls.2024.1391679 (PMC11269227; doi:10.3389/fpls.2024.1391679)
Supplement: Supplementary file 2 [file Image_2.pdf]

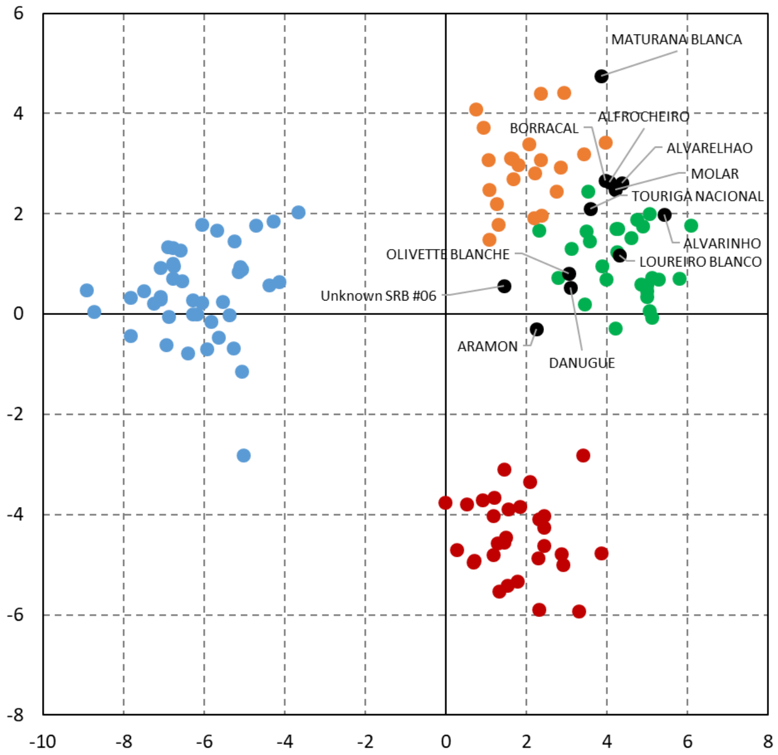

Supplementary Material 7. Discriminant analysis of principal components (DAPC) obtained between the Balkan grapevine varieties identified in this work and some selected varieties of the Caucasus, Central Europe, and the Iberian Peninsula genetic pools. Grapevine varieties are shown as dots, with colours denoting group allocation (group 1: blue; group 2: red; group 3: orange; group 4: green), but for the varieties highlighted in the manuscript, shown as black dots. The origin of the grapevines used in this analysis and their group allocation are indicated in the Supplementary Materials 2 and 3.
